# Ultra-Sensitive Impedimetric Immunosensor Using Copper Oxide Quantum Dots Grafted on the Gold Microelectrode for the Detection of Parathion

**DOI:** 10.3390/mi13091385

**Published:** 2022-08-25

**Authors:** Shalini Nagabooshanam, Bhusankar Talluri, Tiju Thomas, Satheesh Krishnamurthy, Ashish Mathur

**Affiliations:** 1 Department of Mechanical Engineering, Indian Institute of Technology Madras, Chennai 600036, India; 2 Amity Institute of Nanotechnology, Amity University Uttar Pradesh, Sector-125, Noida 201301, India; 3 Department of Metallurgical and Material Engineering, Indian Institute of Technology Madras, Chennai 600036, India; 4 Nanoscale Energy and Surface Engineering School of Engineering and Innovation, The Open University, Milton Keynes MK7 6AA, UK; 5 Department of Physics, University of Petroleum and Energy Studies, Dehradun 248007, India

**Keywords:** organophosphates, immunosensor, quantum dots, copper oxide, electrochemical

## Abstract

The extensive use of organophosphates (OPs) pollutes the environment, leading to serious health hazards for human beings. The current need is to fabricate a sensing platform that will be sensitive and selective towards the detection of OPs at trace levels in the nM to fM range. With this discussed in the present report, an ultra-sensitive immunosensing platform is developed using digestive-ripened copper oxide quantum dots grafted on a gold microelectrode (Au-µE) for the impedimetric detection of parathion (PT). The copper oxide quantum dots utilized in this study were of ultra-small size with a radius of approximately 2 to 3 nm and were monodispersed with readily available functional groups for the potential immobilization of antibody parathion (Anti-PT). The miniaturization is achieved by the utilization of Au-µE and the microfluidic platform utilized has the sample holding capacity of about 2 to 10 µL. The developed immunosensor provided a wide linear range of detection from 1 µM to 1 fM. The lower Limit of Detection (LoD) for the developed sensing platform was calculated to be 0.69 fM, with the sensitivity calculated to be 0.14 kΩ/nM/mm^2^. The stability of the sensor was found to be ~40 days with good selectivity. The developed sensor has the potential to integrate with a portable device for field applications.

## 1. Introduction

Globally, during pre-harvest, almost 35% of typical crop production is lost owing to pests and insects damaging the crops [1]. In agricultural practices, the judicious usage of pesticides and insecticides will help produce a multi-fold increase in crop yield [2]. Regardless of their benefits, the extensive utilization of pesticides has generated chaos for the environment as well as living beings due to serious health hazards. Organophosphates (OPs) are predominantly utilized in agricultural fields compared to other classified pesticides such as carbamates, organochlorines, bio-pesticides and pyrethroids [3]. All OPs are degraded quickly in normal circumstance by oxidation and mainly by hydrolysis, but they do exist in various forms of metabolites which remains as residues in the environment [4]. Several OPs such as parathion, methyl parathion, monocrotophos, chlorpyrifos, etc., cause acute toxicity even at low concentrations (µM to nM). They generate various adverse health effects such as neurological disorders, genotoxicity, respiratory disorders, chronic kidney disease, cancer, poisoning, reproductive disruption, etc. [5].

Widely used chromatographic methods such as gas chromatography (GC), liquid chromatography (LC), mass spectrometry (MS), capillary electrophoresis (CE), ion mobility spectrometry (IMS), gas chromatography-mass spectrometry (GC-MS), liquid chromatography-mass spectrometry (LC-MS) and high-performance liquid chromatography (HPLC) have traditionally been used for the detection of OPs. Other techniques include spectrophotometric and immunochemical sensors [6]. The aforementioned techniques have major drawbacks such as cumbersome sample separation process, device complexity, co-elution as in HPLC, longer time periods for sample preparation and the separation process, expensive reagents usage, huge sample volumes and high analysis cost. The essential need is to develop a sensor having high selectivity, high sensitivity, low detection limits, wide detection range, good economy, easily operation and does not require a sophisticated analytical laboratory. The aforementioned needs can be satisfied by the development of bio-sensors.

Biosensors are chosen as the best alternative over conventional techniques due to their ease of use and their economical, selective, and sensitive platform. Biosensors are molecular analytical devices that provide a detectable biochemical or physiochemical signal upon the biological interaction between the analyte and receptor of interest [7]. Various bio-receptors are available for the sensitive detection of OPs but, among them, immunosensors are more versatile in terms of selectivity and sensitivity [8]. Combining immunosensors with Electrochemical Impedance Spectroscopy (EIS), which is the most promising electrochemical technique, will provide a better sensing platform for the direct sensing of analytes based on the simple affinity complex formation [9,10].

Various metal oxides, nanomaterials and quantum dots having uniform size, morphology and functional groups are extensively studied for various applications [11,12,13,14]. Among them, copper oxide quantum dots are most appropriate owing to their excellent conductivity, electrocatalytic activity and electronic properties which have been well explored for many applications including sensing [15,16]. These copper oxide quantum dots can be synthesized by many routes to obtain monodispersed and highly stable materials which help in enhancing sensing properties [17]. Thus far, the digestively ripened method has provided monodispersed and highly stable quantum dots, while the digestive ripening agent utilized also provides various functional groups which help in efficient biomolecule immobilization [18]. In this present study, an impedimetric immunosensor was developed to detect PT using the monodispersed copper oxide quantum dots.

## 2. Experimental

### 2.1. Chemicals and Methods

Anti-parathion (Anti-PT), parathion (PT), chlorpyrifos (CPF), monocrotophos (MCP), malathion (MT) and bovine serum albumin (BSA) was bought from Sigma-Aldrich. Sodium chloride (NaCl), potassium ferricyanide (K_3_[Fe(CN)_6_]), potassium ferrocyanide (K_4_[Fe(CN)_6_]), monobasic (NaH_2_PO_4_.2H_2_O) and dibasic (Na_2_HPO_4_) were purchased from Fisher Scientific. Copper acetate monohydrate (Cu(OAc)_2_H_2_O), sodium hydroxide (NaOH), triethanolamine (TEA) and ethanol were obtained from Merck. Throughout the experiments, Milli-Q deionized (D.I) water (18.2 MΩ) and analytical grade reagents were utilized. Gold micro-electrodes (Au-µE) and microfluidic cell platform (sample consumption of about 2–10 µL) were obtained from MicruX Tech., Spain [19]. The overall dimension of the Au-µE was 10 × 6 × 0.75 mm and the radius of the working electrode was 0.5 mm. All electrochemical experiments were performed in phosphate buffer saline (PBS) (0.1 M, pH7) containing the redox probe (5 mMferri/ferro). The electrodes were cleaned by sonication for 5 min in acetone and 5 min in D.I. water prior to use. All experiments were performed three times and the average was plotted with error bars from the standard deviation (*n* = 3).

### 2.2. Instruments

Electrochemical impedance spectroscopy (EIS) was performed using a Multiautolab (MA204) potentiostat/galvanostat (The Netherlands). FTIR spectroscopy was performed using a Frontier Perkin Elmer infrared spectrophotometer. X-ray diffraction patterns were obtained on a PAN analytical X’Pert PRO spectrometer using Cu-K_α_ source (λ = 0.154 nm). Transmission electron micrographs images were acquired using a Philips FEI TECNAI G2S TEM operating at 200 kV.

### 2.3. Synthesis of Copper Oxide Quantum Dots

Copper (II) oxide quantum dots (CuO) were synthesized using the following protocol by the digestive ripening method [16]. A 3 mM concentration of copper acetate monohydrate in ethanol solution was prepared. The addition of 18 mM sodium hydroxide (solid) to the reaction mixture then facilitated the growth of copper oxide. The whole synthesis was carried out at room temperature with continuous vigorous stirring at 1100 to 1200 rpm. Once the sodium hydroxide was completely dissolved, 30 mM TEA was added in a dropwise manner to the above reaction mixture, leading to the formation of digestively ripened copper oxide quantum dots.

### 2.4. Development of Anti-PT/CuO/Au-µE

**Figure 1:**
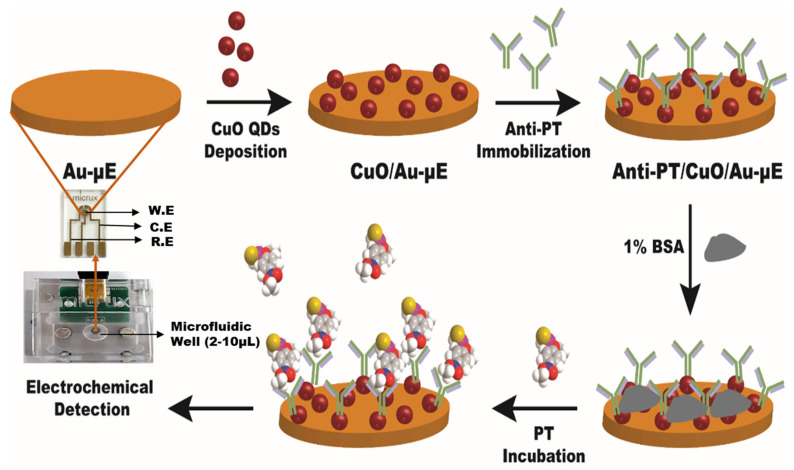
Graphical representation of successive fabrication steps of Anti-PT/CuO/Au-µE (W.E—working electrode; C.E—counter electrode; R.E—reference electrode).

A graphical representation of successive fabrication steps of Anti-PT/CuO/Au-µE is shown in Figure 1. In step one, the CuO quantum dots (2 µL) were coated on the Au-µE by the drop casting technique. The electrode was then dried for 20 min at ambient room temperature. In step two, the antibody Anti-PT was immobilized on the CuO modified electrode and incubated for 30 min at 37 °C. The Anti-PT/CuO/Au-µE was washed with a copious amount of PBS buffer solution in order to remove the unbound Anti-PT antibodies. The unspecific sites were blocked with 1% BSA solution and washed three times with PBS buffer solution for removing unbound BSA. The final electrode Anti-PT/CuO/Au-µE was stored at 4 °C for use in further experiments.

## 3. Results and Discussion

### 3.1. Characterization of Synthesized Copper Oxide Quantum Dots

**Figure 2:**
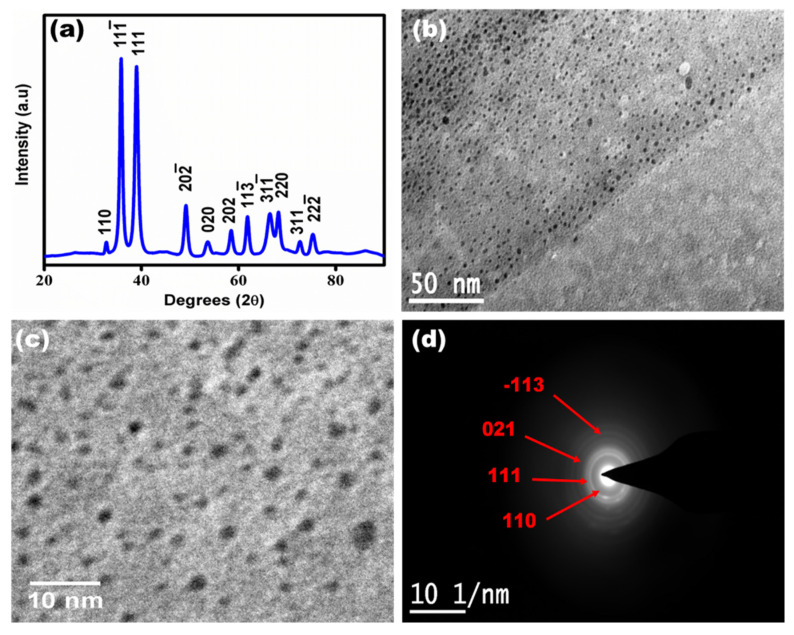
(**a**) XRD analysis for the synthesized copper oxide quantum dots, (**b**) HR-TEM micrograph of CuO QDs at 50 nm scale bar, (**c**) HR-TEM micrograph of CuO QDs at 10 nm scale bar and (**d**) electron diffraction pattern of CuO QDs showing the cupric oxide phase formation at 1/10 nm scale.

The synthesized copper oxide quantum dots were characterized using XRD and HR-TEM as shown in Figure 2. Figure 2a shows the XRD analysis of the CuO QDs. It was observed that the two prominent pointed peaks at 2θ = 35.6 and 38.9 reflections attributed to the (−111) and (111) planes of CuO having monoclinic structure, which is in agreement with ICSD No. 98-001-7494. The XRD analysis confirmed the formation of cupric oxide (Cu(II)O) phase with a monoclinic crystal structure, consistent with previous reports [20,21]. Figure 2b,c shows a bright-field TEM image at 50 nm and 10 nm scale bar of CuO QDs having quasi-spherical morphology without agglomeration [22]. The TEM micrographs indicate the formation of highly monodispersed quantum dots with an average particle size ~2.4 ± 0.5 nm. An electron diffraction pattern of CuO QDs is shown in Figure 2d, confirming the formation of the cupric oxide phase with rings indexed at planes (111) [23].

### 3.2. Characterization of the Developed Immunosensing Platform (Anti-PT/CuO/Au-µE)

**Figure 3:**
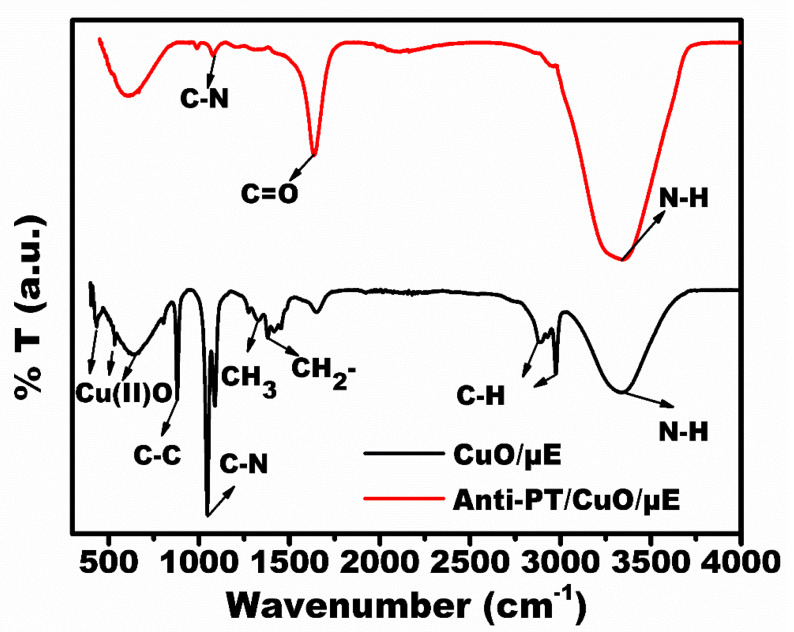
FTIR spectral analysis of the developed sensor (Anti-PT/CuO/Au-µE).

FTIR spectral analysis was performed to check the successful modification at each stage as shown in Figure 3. The CuO/Au-µE spectra are shown in black color. The bands at 435 cm^−1^, 639 cm^−1^ and 535 cm^−1^ are attributed to the presence of cupric oxide phase [24]. The bands at 2879 cm^−1^ and 2980 cm^−1^ were attributed to C-H vibrations [25]. The spectral bands at 1379 cm^−1^ and 895 cm^−1^ correspond to the stretching vibrations of CH_2_ and C-C. The bands at 1054 cm^−1^ and 3339 cm^−1^ correspond to C-N and N-H vibrations from the TEA [26]. Upon the immobilization of the Anti-PT antibody, the strong spectral bands at 1637 cm^−1^ and 3320 cm^−1^ were directly attributed to the amide bond formation by C=O and N-H bonds [27]. The weak C-N band at 1053 cm^−1^ confirms the effective immobilization of Anti-PT onto CuO/Au-µE.

**Figure 4:**
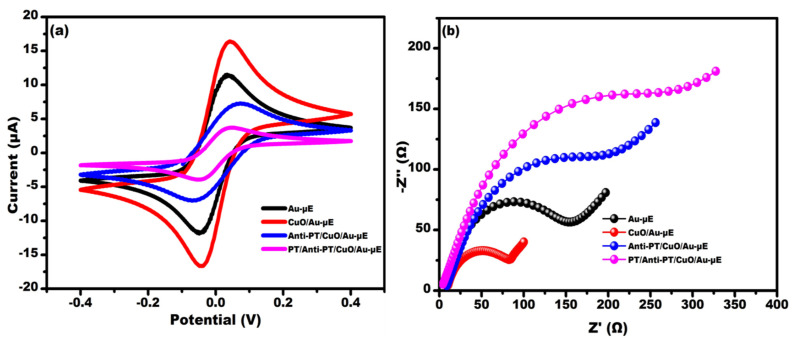
Electrochemical characterization of the fabricated sensor at each successive modification of electrode by (**a**) CV and (**b**) EIS.

Figure 4 shows the electrochemical characterization at various stages of surface modification recorded using CV and EIS. Figure 4a shows the cyclic voltammogram response recorded within the potential −0.4 V to +0.4 V scanned at 50 mV/s in 0.1 M PBS buffer containing 5 mM redox probe (ferro/ferri solution). It was observed that the maximum current response (16.7 µA) obtained for CuO/Au-µE compared to Au-µE (11.4 µA) due to the excellent conducting property of CuO QDs. The current response decreased to 6.9 µA upon the immobilization of Anti-PT antibody, which is a consequence of the insulating nature of the biomolecules. After the immune-complex formation, the current response decreased to 3.5 µA due to the interfacial charge transfer hindrance between the electrode surface and the electrolyte.

Figure 4b shows the Nyquist spectra recorded in the frequency range of 100 Hz to 50 kHz at an ac amplitude of 10 mV in the presence of 0.1 M PBS buffer containing 5 mM redox probe. The Nyquist spectra clearly depict a semi-circular feature corresponding to the simultaneous occurrence of charge transfer resistance (Rct) and the formation of double layer capacitance at higher frequencies and a Warburg diffusion parameter at lower frequencies. Insights into these interfacial parameters involved at various stages of sensor fabrication were obtained through simulation and fitting of Nyquist spectra using the simulation and fitting software Nova 1.11. A Randles equivalent circuit was used to model the different interfaces during sensor fabrication. Nova 1.11 software was utilized for fitting and simulation. It was observed that for CuO/Au-µE, a lower charge transfer resistance (Rct) value of 81 Ω was obtained in comparison to Au-µE having a Rct value of 152 Ω, which is attributed to the excellent conducting property of CuO QDs. The Rct increased to 194 Ω upon the immobilization of Anti-PT antibody, which is a consequence of the insulating nature of the biomolecules. After the immune-complex formation, the Rct increased to 274 Ω due to the interfacial charge transfer hindrance between the electrode and electrolyte.

### 3.3. Impedimetric Detection of Parathion

**Figure 5:**
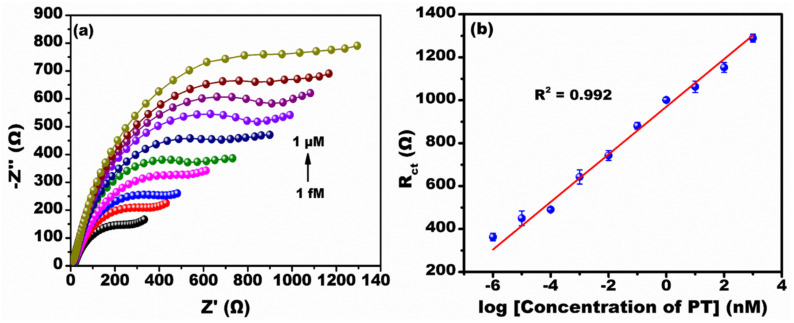
Electrochemical detection of PT by EIS. (**a**) Nyquist spectra at various concentrations (1 fM to 1 µM) of PT; (**b**) The calibration plot at various log concentrations of PT.

Figure 5a shows the Nyquist spectra recorded at various concentration ranges of PT (1 fM to 1 µM) in the frequency range of 100 Hz to 50 kHz at an ac amplitude of 10 mV measured in the presence of 0.1 M PBS buffer. The Nyquist spectra show typical semi-circular features corresponding to charge transfer resistance and the Faradaic process of interfacial charge transfer [28]. It was observed that as the concentration of PT increased, the Rct value increased linearly. This situation is ascribed to the formation of an immune-complex reaction between the PT antibody and PT antigen resulting in increased Rct values. Figure 5b shows the calibration curve at different concentrations of PT from 1 fM to 1 µM. It was observed that the Rct is directly proportional to the log of the concentration of PT. The linear regression equation from the calibration curve was generated by the intercept and slope values. The calculated intercept and slope values were approximately 969.79 ± 14.14 and 110.94 ± 4.41, respectively, with line equation y = 110.94x + 969.79 (x = log concentration of PT (nM)) and coefficient r^2^ = 0.99. The developed immunosensor provided a linear detection range of 1 fM to 1 µM. The calculated LoD from the 3σ rule was about 0.69 fM [29] and the sensitivity was calculated to be 0.14 kΩ/nM/mm^2^ [30].

### 3.4. Selectivity and Stability Analysis

**Figure 6:**
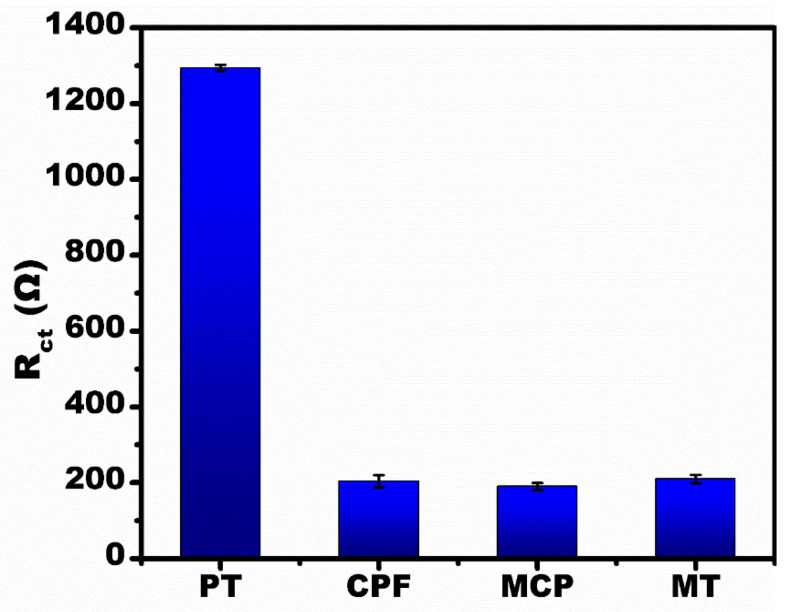
Selectivity studies for the developed sensor at 1 µM concentration of various OPs (PT, CPF MT, MCP).

Figure 6 shows the selectivity of the fabricated sensor. To study the selectivity, 1 µM solutions of PT, CPF, MT and MCP were prepared. The R_ct_ value for CPF, MT and MCP was approximately a similar value to the control sample (i.e., Anti-PT/CuO/Au-µE; for reference, see Figure 4b). For Anti-PT specific antigen PT, the R_ct_ value was elevated due to the explicit binding at the affinity sites. The drastic increase in the R_ct_ value only for the PT in the mixture solution containing CPF, MT and MCP is evidence of the selective binding efficacy of the developed sensor.

**Figure 7:**
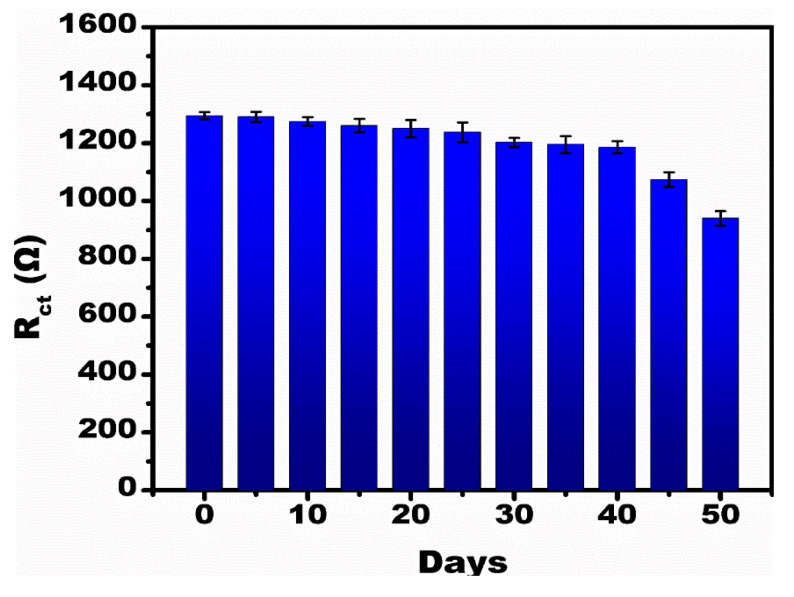
Stability analysis for the developed sensor at 1 µM PT concentration tested at intervals of 5 days up to 50 days.

Figure 7 shows the stability analysis of the developed sensor. To test the stability, identical multiple electrodes were prepared. The prepared electrodes were tested at 1 µM concentration of PT of 50 days with an interval of 5 days. It was observed that till 40 days there was no major change in the R_ct_ value, but after 40 days a >15% decrease in the Rct value signifies that the sensor performance in detecting PT is stable for up to 40 days. The developed sensor compared to other reported impedimetric immunosensors for the detection of PT is shown in Table 1.

## 4. Conclusions

The copper oxide quantum dots synthesized by the digestive ripening route provided better uniform size and high stability. The readily available functional groups on the copper oxide quantum dots provided good surface chemistry for the covalent immobilization of antibodies. The attractive electrocatalytic property of copper oxide enhanced the electrochemical sensing performance, which provided a wide linear detection range from 1 fM to 1 µM with LoD of approximately 0.69 fM and sensitivity of 0.14 kΩ/nM/mm^2^. The developed immunosensor showed excellent sensitivity with a lower limit of detection compared to other reported sensors. The developed sensor showed stability of 40 days and excellent specificity to PT and has the capability for field trials by integrating it to portable electronics.

## Figures and Tables

**Table 1:** Comparison of other sensors reported for the sensing of PT.

Senor	Technique	Linear Range	LoD	Reference
[Cd(atc)(H_2_O)_2_]n/ITO	Impedimetric	0.1 to 20 ng/mL	0.1 ng/mL	[27]
AntiPT/2ABA/Graphene/SPE	Impedimetric	0.1 to 1000 ng/L	52 pg/L (17.8 fM)	[31]
GQD/SPE	Impedimetric	0.01 to 106 ng/L	46 pg/L (15.8 fM)	[32]
Anti-PT/CuO/Au-µE	Impedimetric	1 fM to 1 µM	0.69 fM	Present Work

atc-2—aminoterephthalic acid; ITO—indium tin oxide; 2ABA—2,aminobenzyl amine; SPE—screen-printed electrode; GQD—graphene quantum dot.
